# Supplementary material for: The APOE isoforms differentially shape the transcriptomic and epigenomic landscapes of human microglia xenografted into a mouse model of Alzheimer’s disease
Source: Nat Commun. 2025 May 27;16:4883. doi: 10.1038/s41467-025-60099-4 (PMC12106835; doi:10.1038/s41467-025-60099-4)
Supplement: Supplementary file 1 — Supplementary Information [file 41467_2025_60099_MOESM1_ESM.pdf]

Supplementary Information

The APOE isoforms differentially shape the transcriptomic and epigenomic landscapes of human microglia in a xenotransplantation model of Alzheimer's disease

Kitty B. Murphy, Di Hu, Leen Wolfs, Susan K. Rohde, Gonzalo Leguía Fauró, Ivana Geric, Renzo Mancuso, Bart De Strooper and Sarah J. Marzi

Supplementary Figures

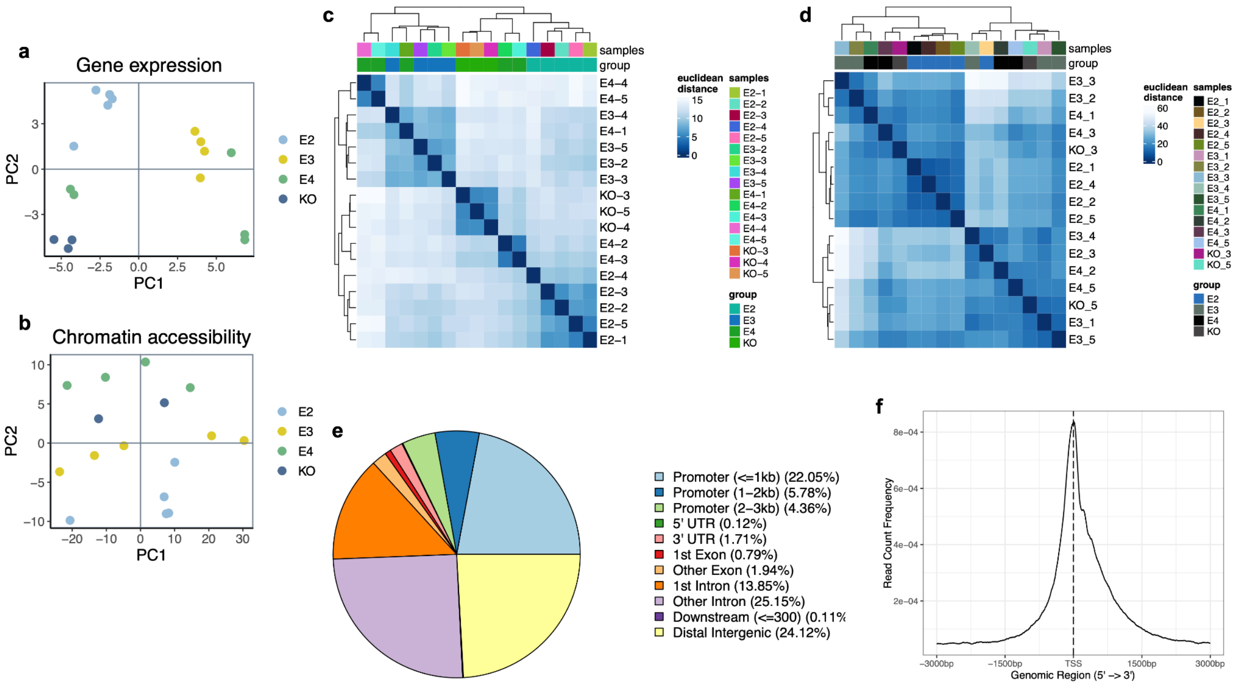

Supplementary Figure 1: QC of ATAC-seq and RNA-seq samples. a PCA on gene expression across the APOE isoforms and the APOE-KO. Sample size: *APOE2* = 5, *APOE3* = 4, *APOE4* = 5, *APOE*-KO = 3. PCA was generated using the variance stabilising transformation (VST)-normalised expression counts matrix. **b** PCA on genome-wide chromatin accessibility across the APOE isoforms and the APOE-KO. Sample size: *APOE2* = 5, *APOE3* = 5, *APOE4* = 4, *APOE*-KO = 2. PCA was generated using the variance stabilising transformation (VST)-normalised expression counts matrix. **c** Hierarchical clustering using euclidean distance of VST-normalised expression counts of the APOE isoforms and the APOE-KO. **d** Hierarchical clustering using euclidean distance of VST-normalised ATAC-seq read counts of the APOE isoforms and the APOE-KO. **e** Pie chart of genomic annotations of the consensus set of chromatin accessibility peaks. **f** Transcription start site (TSS) enrichment profiles of ATAC-seq peaks. Source data are provided as a Source Data file.

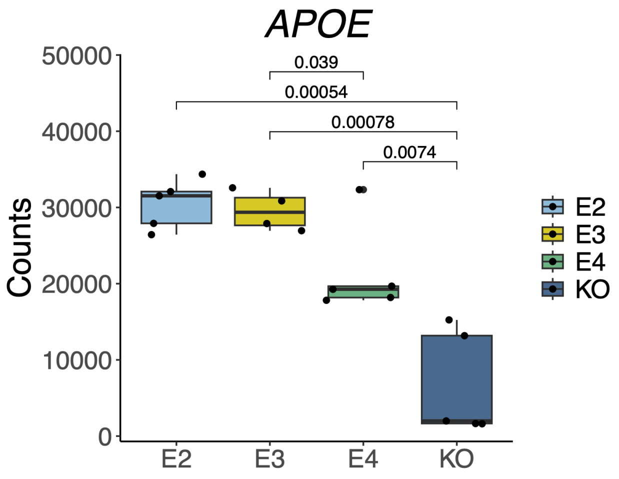

Supplementary Figure 2: Boxplot of gene expression profiles of *APOE*, including two excluded *APOE*-KO samples which did not show loss of *APOE* expression. Sample size: *APOE2* = 5, *APOE3* = 4, *APOE4* = 5, *APOE*-KO = 5. The two-sided Wilcoxon rank-sum test was used to calculate p-values. The central mark and edges indicate the 50th (median), 25th and 75th percentiles. Whiskers correspond to 1.5 * the IQR. Source data are provided as a Source Data file.

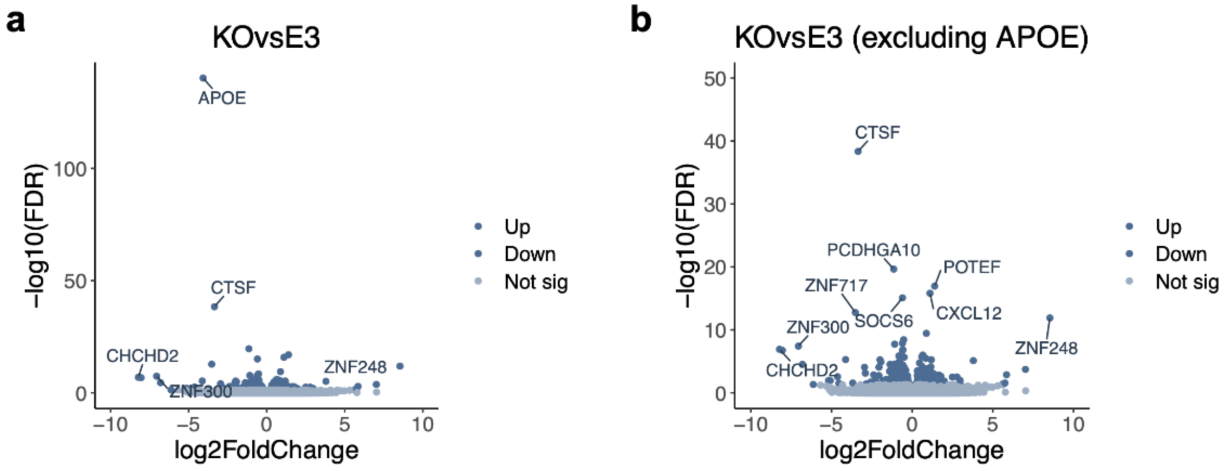

Supplementary Figure 3: Differentially expressed genes in *APOE4* compared to the KO. Differentially expressed genes in **a** *APOE* KO vs *APOE3*, **b** *APOE* KO vs *APOE3*, excluding *APOE* in the plot. Sample size: *APOE2* = 5, *APOE3* = 4, *APOE4* = 5, *APOE*-KO = 3. Source data are provided as a Source Data file.

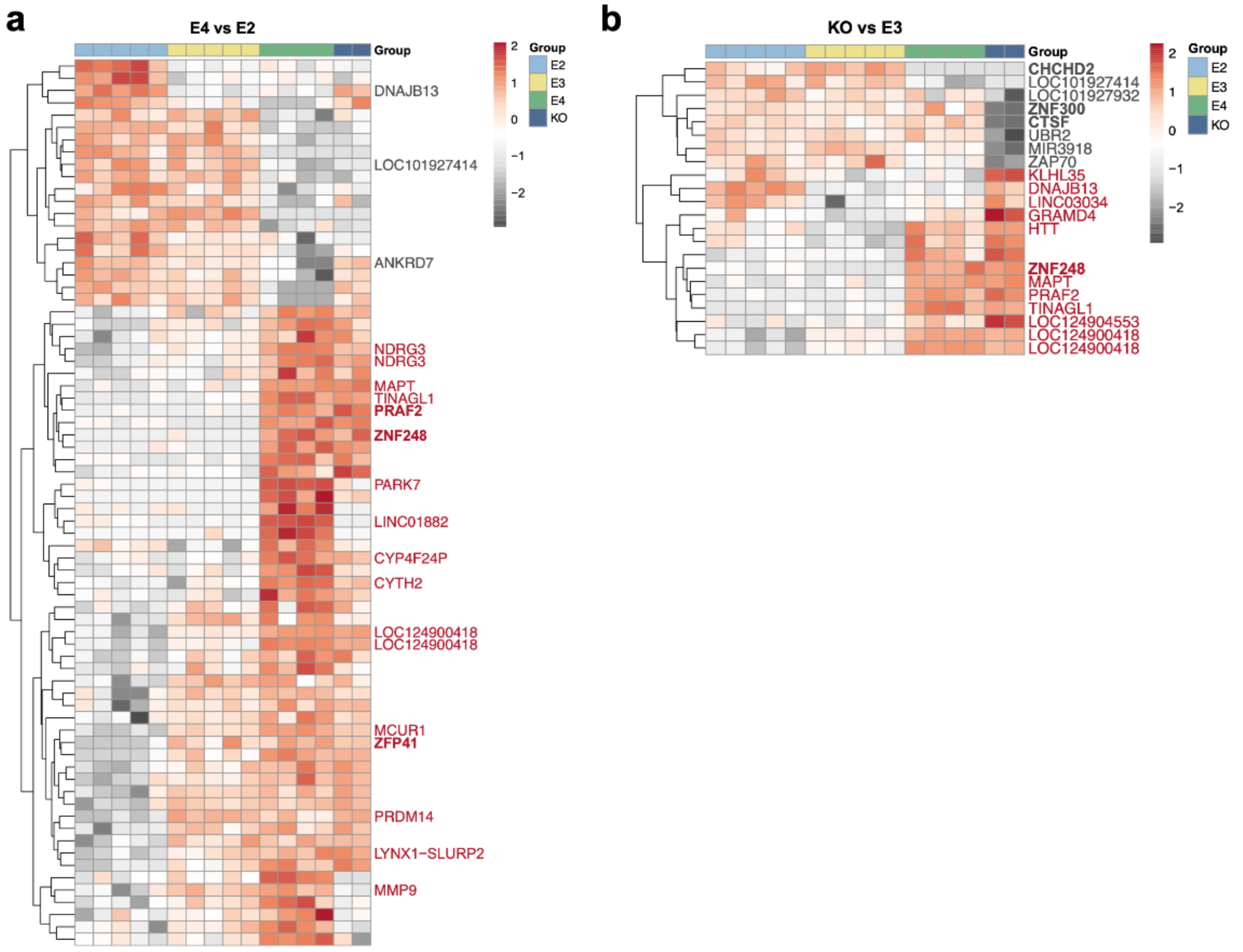

Supplementary Figure 4: Differentially accessible regions are annotated to differentially expressed genes. Heatmaps showing differential accessibility of significant peaks (FDR < 0.05) when comparing **a** *APOE4* vs *APOE2*, **b** *APOE*-KO vs *APOE3*. Sample size: *APOE2* = 5, *APOE3* = 5, *APOE4* = 4, *APOE*-KO = 2. Shown are the genes annotated to the top 20 peaks, genes marked in bold were also significantly differentially expressed in the RNA-seq analysis. Source data are provided as a Source Data file.

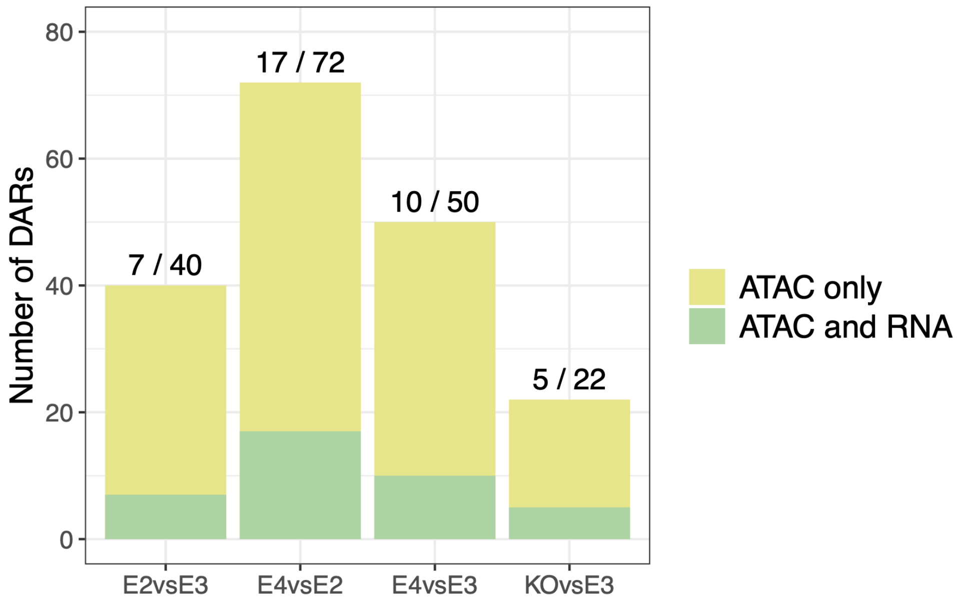

Supplementary Figure 5: Number of differentially accessible regions and how many are annotated to differentially expressed genes. Stacked barplot of the number of differentially accessible regions (DARs) and how many overlap with the DEGs based on peak-to-gene annotation. Sample size: *APOE2* = 5, *APOE3* = 4, *APOE4* = 5, *APOE*-KO = 3. Source data are provided as a Source Data file.

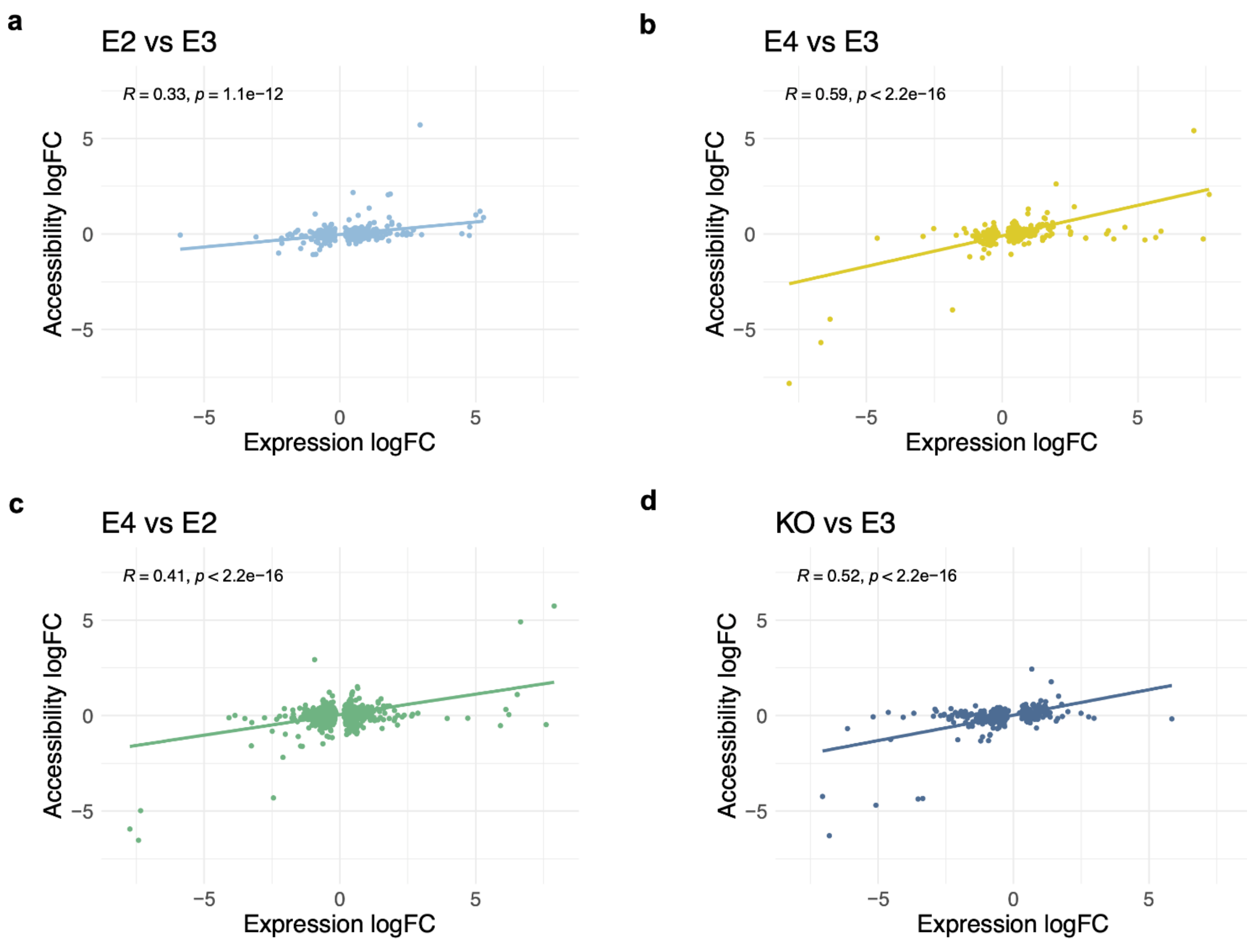

Supplementary Figure 6: Correlation of the expression and chromatin accessibility logFC. RNA-seq logFC and ATAC-seq logFC correlate across DEGs and their promoter peaks in **a** *APOE2* vs *APOE4*, **b** *APOE4* vs *APOE3*, **c** *APOE4* vs *APOE2*, **d** *APOE* KO vs *APOE3*. Correlations were performed using Pearson's product moment correlation coefficient. Source data are provided as a Source Data file.

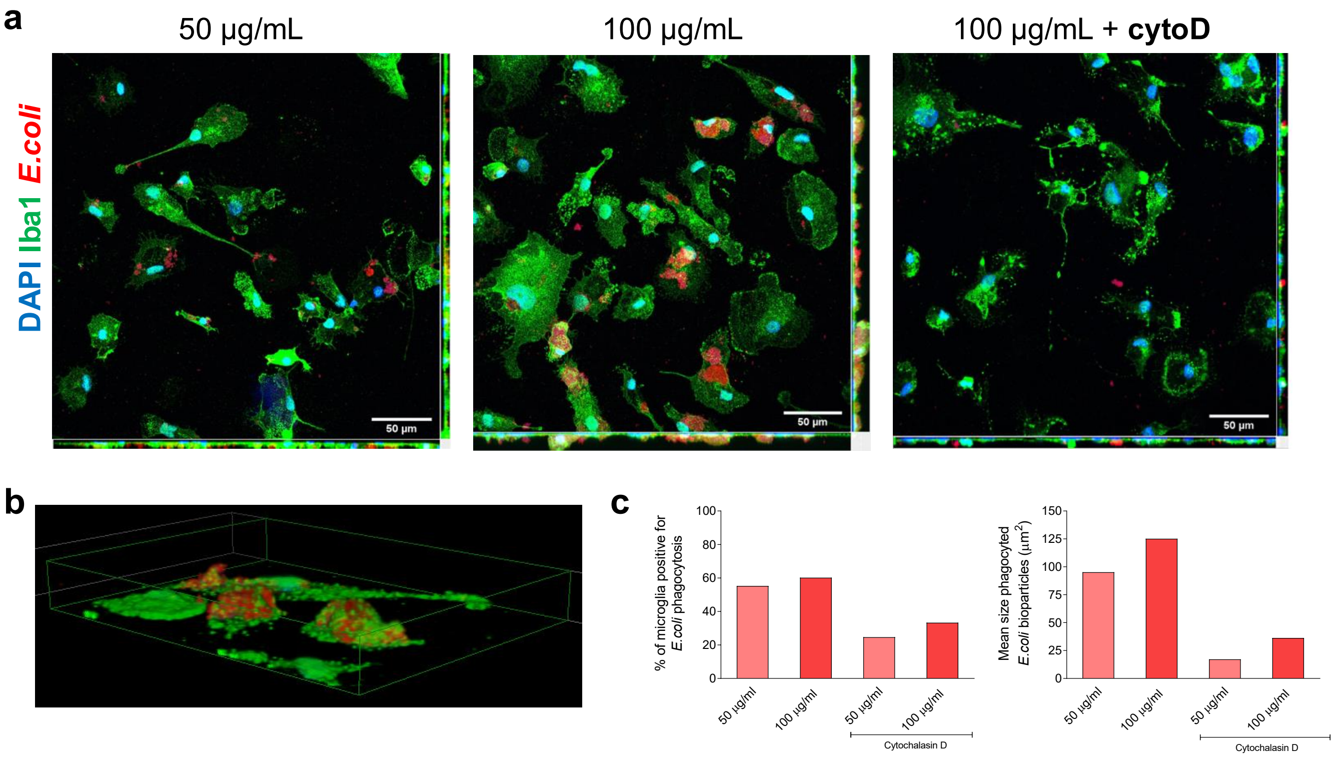

Supplementary Figure 7. Confirmation of pHrodo E. coli particles update by iPSC-derived microglia. a Representative images of pHrodo E. Coli particles at 50 and 100ug/ml. **b** 3D reconstruction showing that the particles quantified are inside the microglial cytoplasm. **c** Quantification of the % of cells showing pHrodo signal and the mean size of intracellular pHrodo particles per cell. Addition of cytochalasin D (Cyto D) was used to confirm that the observed intracellular signal was due to uptake. Source data are provided as a Source Data file.

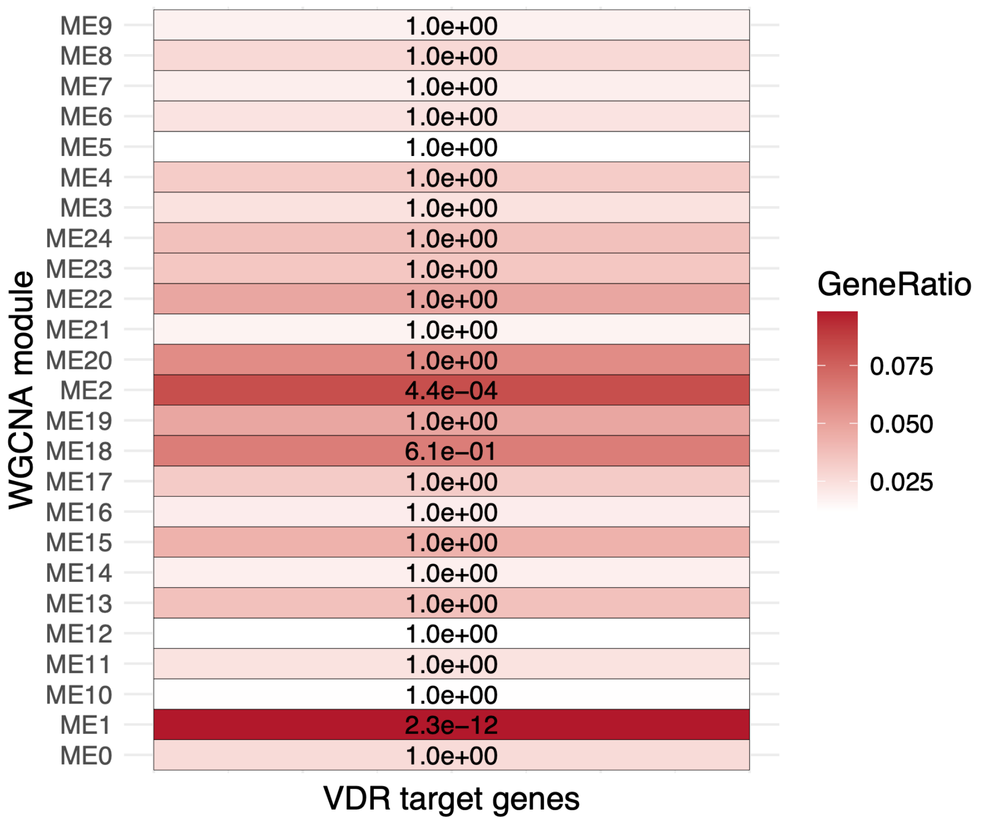

Supplementary Figure 8: VDR target genes are enriched in a gene module upregulated in *APOE2*-expressing microglia. VDR target genes were enriched in two WGCNA modules identified in this study. The ME1 (dark green) module, which we functionally characterised as an immune response module (Fig. 5 d,e), and the ME2 (blue) module, which was not differentially expressed across any of the APOE isoforms (FDR < 0.05). P-values were computed using a one-sided hypergeometric test. Source data are provided as a Source Data file.

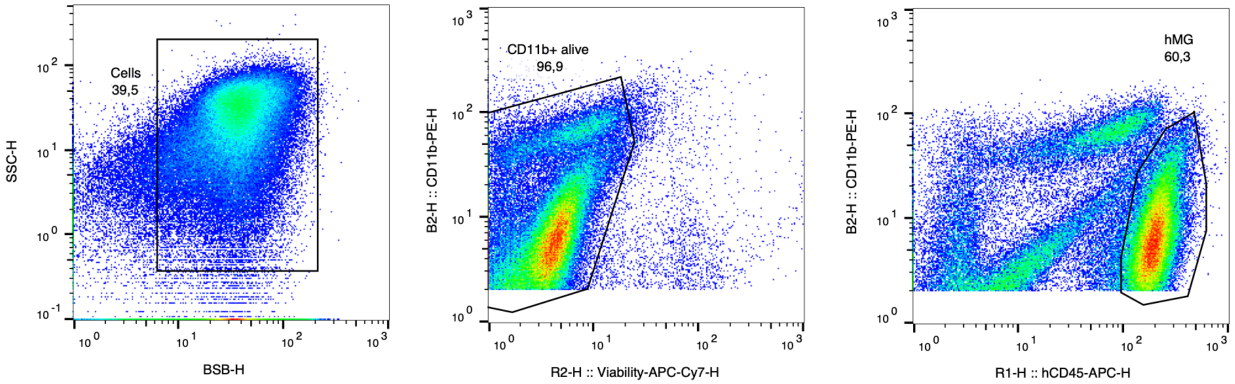

Supplementary Figure 9: Gating strategy for the sorting of xenotransplanted human microglia from the mouse brain. Human microglia ($CD11b^+$ $hCD45^+$) were isolated using the MACSQuant Tyto system, with CD11b set as the fluorescence trigger and hCD45 as the gating marker to define positive events. Due to the system's trigger-based sorting mechanism, $CD11b^-$ events were excluded from visualisation during sorting. This strategy, based on cell speed and fluorescence trigger, was consistent with the approach used in Mancuso et al. 2024, where updated software enabled visualisation of negative populations. The sorted population is outlined and depicted as hMG. Source data are provided as a Source Data file.
