# Supplementary material for: The APOE isoforms differentially shape the transcriptomic and epigenomic landscapes of human microglia xenografted into a mouse model of Alzheimer’s disease
Source: Nat Commun. 2025 May 27;16:4883. doi: 10.1038/s41467-025-60099-4 (PMC12106835; doi:10.1038/s41467-025-60099-4)
Supplement: Supplementary file 2 — Description of Additional Supplementary Files [file 41467_2025_60099_MOESM2_ESM.pdf]

Description of Additional Supplementary Files

Supplementary Data 1. QC metrics for the RNA-seq and ATAC-seq data.

Supplementary Data 2. DESeq2 differential expression analysis results. Results are provided for the following comparisons: *APOE2* vs *APOE3*, *APOE4* vs *APOE3*, *APOE4* vs *APOE2*, *APOE*-KO vs *APOE3*. The nbinomWaldTest() called within the DESeq() function fits a negative binomial generalised linear model and performs a two-sided Wald test. P-values were corrected using the FDR.

Supplementary Data 3. MAGMA gene set analysis. CSV file containing MAGMA gene set analysis results using the differentially expressed genes (FDR < 0.05) for the comparisons outlined in Supplementary Table 2 and three independent AD GWAS.

Supplementary Data 4. DESeq2 differential accessibility analysis results. Results are provided for the following comparisons: *APOE2* vs *APOE3*, *APOE4* vs *APOE3*, *APOE4* vs *APOE2*, *APOE*-KO vs *APOE3*. The nbinomWaldTest() called within the DESeq() function fits a negative binomial generalised linear model and performs a two-sided Wald test. P-values were corrected using the FDR.

Supplementary Data 5. Linkage disequilibrium score regression. CSV file containing s-LDSC results using the consensus set of ATAC-seq peaks with three brain disorder GWAS (Alzheimer's disease, autism spectrum disorder, and amyotrophic lateral sclerosis).

Supplementary Data 6. Pathway enrichment of WGCNA modules. CSV file containing pathway enrichment results using two WGCNA-identified modules significantly upregulated in *APOE2*-expressing microglia. P-values were computed using a one-sided Fisher's exact test.

Supplementary Data 7. Transcription factor motif enrichment results. Homer motif enrichment analysis results using top 100 peaks with increased and decreased chromatin

accessibility for *APOE2* vs *APOE3*, *APOE4* vs *APOE3*, and *APOE4* vs *APOE2*. P-values were computed using a one-sided hypergeometric test.

All supplementary data files are available on Zenodo: 10.5281/zenodo.12516685.
