# Supplementary material for: The APOE isoforms differentially shape the transcriptomic and epigenomic landscapes of human microglia xenografted into a mouse model of Alzheimer’s disease
Source: Nat Commun. 2025 May 27;16:4883. doi: 10.1038/s41467-025-60099-4 (PMC12106835; doi:10.1038/s41467-025-60099-4)
Supplement: Supplementary file 10 — Reporting Summary [file 41467_2025_60099_MOESM10_ESM.pdf]

nature portfolio

Corresponding author(s): Sarah J Marzi

Last updated by author(s): Apr 3, 2025

Reporting Summary

Nature Portfolio wishes to improve the reproducibility of the work that we publish. This form provides structure for consistency and transparency in reporting. For further information on Nature Portfolio policies, see our Editorial Policies and the Editorial Policy Checklist.

Statistics

For all statistical analyses, confirm that the following items are present in the figure legend, table legend, main text, or Methods section.

n/a	Confirmed	
[ ]	[x]	The exact sample size (*n*) for each experimental group/condition, given as a discrete number and unit of measurement
[ ]	[x]	A statement on whether measurements were taken from distinct samples or whether the same sample was measured repeatedly
[ ]	[x]	The statistical test(s) used AND whether they are one- or two-sided<br>*Only common tests should be described solely by name; describe more complex techniques in the Methods section.*
[ ]	[x]	A description of all covariates tested
[ ]	[x]	A description of any assumptions or corrections, such as tests of normality and adjustment for multiple comparisons
[ ]	[x]	A full description of the statistical parameters including central tendency (e.g. means) or other basic estimates (e.g. regression coefficient) AND variation (e.g. standard deviation) or associated estimates of uncertainty (e.g. confidence intervals)
[ ]	[x]	For null hypothesis testing, the test statistic (e.g. *F*, *t*, *r*) with confidence intervals, effect sizes, degrees of freedom and *P* value noted<br>*Give P values as exact values whenever suitable.*
[x]	[ ]	For Bayesian analysis, information on the choice of priors and Markov chain Monte Carlo settings
[x]	[ ]	For hierarchical and complex designs, identification of the appropriate level for tests and full reporting of outcomes
[ ]	[x]	Estimates of effect sizes (e.g. Cohen's *d*, Pearson's *r*), indicating how they were calculated

Our web collection on statistics for biologists contains articles on many of the points above.

Software and code

Policy information about availability of computer code

Data collection	RNA-seq and ATAC-seq data were generated from human microglia isolated from a microglia xenotransplantation model of Alzheimer's disease. No software or code was used in the collection of these data.
Data analysis	General QC: FASTQC<br>Alignment: bowtie2 v2.4.2<br>Sorting and indexing: samtools v1.9<br>Removal of duplicates: picard (JAVA version 21)<br>Peak calling: MACS3 v3.0.0b3<br>Filtering (non-uniquely mapped reads, quality threshold): samtools v1.9<br>Filtering of blacklist regions: bedtools v2.31.0<br>Read count generation: featureCounts v2.0.3<br>Differential expression and chromatin accessibility analysis: DESeq2 v1.44<br>Peak annotation: ChIPseeker v1.40<br>Pathway enrichment analysis: clusterProfiler v4.12<br>Motif enrichment analysis: HOMER v4.11<br>Weighted gene co-expression network analysis (WGNA): v1.73<br>LDSC: v1.0.1

For manuscripts utilizing custom algorithms or software that are central to the research but not yet described in published literature, software must be made available to editors and reviewers. We strongly encourage code deposition in a community repository (e.g. GitHub). See the Nature Portfolio guidelines for submitting code & software for further information.

Data

Policy information about availability of data

All manuscripts must include a data availability statement. This statement should provide the following information, where applicable:

- Accession codes, unique identifiers, or web links for publicly available datasets
- A description of any restrictions on data availability
- For clinical datasets or third party data, please ensure that the statement adheres to our policy

FASTQ and read count files have been deposited in the Gene Expression Omnibus (GEO) under accession GSE271384 for the ATAC-seq dataset and GSE271385 for the RNA-seq dataset. All the data and code required to reproduce the figures in this manuscript are available in our GitHub repository: https://github.com/neurogenomics/APOE_microglia. All supplementary tables are available at: 10.5281/zenodo.12516685

Research involving human participants, their data, or biological material

Policy information about studies with human participants or human data. See also policy information about sex, gender (identity/presentation), and sexual orientation and race, ethnicity and racism.

Reporting on sex and gender	No human participants were used in this study.
Reporting on race, ethnicity, or other socially relevant groupings	n/a
Population characteristics	n/a
Recruitment	n/a
Ethics oversight	n/a

Note that full information on the approval of the study protocol must also be provided in the manuscript.

Field-specific reporting

Please select the one below that is the best fit for your research. If you are not sure, read the appropriate sections before making your selection.

☒ Life sciences ☐ Behavioural & social sciences ☐ Ecological, evolutionary & environmental sciences

For a reference copy of the document with all sections, see nature.com/documents/nr-reporting-summary-flat.pdf

Life sciences study design

All studies must disclose on these points even when the disclosure is negative.

Sample size	Sample size was estimated based on previous experiments performed (Mancuso et al., 2019; Sala-Frigerio et al. 2019)
Data exclusions	2 APOE-KO samples were excluded due to having high APOE expression based on counts. 1 APOE-KO sample and 1 APOE4 sample was excluded from the ATAC-seq dataset due to not passing QC. 1 One APOE3 sample was discarded before sequencing due to its RNA quality as assessed using Agilent High Sensitivity DNA Kit (5067-2646).
Replication	Final sample numbers for ATAC-seq: APOE2 = 5, APOE3 = 5, APOE4 = 4, APOE-KO = 2; and RNA-seq: APOE2 = 5, APOE4 = 4, APOE4 = 5, APOE-KO = 3.
Randomization	Experimental groups were randomised to avoid litter and cage effects.
Blinding	Investigators were blinded when performing all experiments.

Reporting for specific materials, systems and methods

We require information from authors about some types of materials, experimental systems and methods used in many studies. Here, indicate whether each material, system or method listed is relevant to your study. If you are not sure if a list item applies to your research, read the appropriate section before selecting a response.

Materials & experimental systems

n/a	Involved in the study
☐	☒ Antibodies
☐	☒ Eukaryotic cell lines
☒	☐ Palaeontology and archaeology
☐	☒ Animals and other organisms
☒	☐ Clinical data
☒	☐ Dual use research of concern
☒	☐ Plants

Methods

n/a	Involved in the study
☒	☐ ChIP-seq
☐	☒ Flow cytometry
☒	☐ MRI-based neuroimaging

Antibodies

Antibodies used	For flow cytometry:<br>Mouse FcR blocker (1:10, Miltenyi, Cat#130-092-575)<br>PE-Pan-CD11b (1:50, Miltenyi, Cat#130-113-806)<br>BV421-mCD45 (1:500, BD Biosciences, Cat#563890)<br>APC-hCD45 (1:50, BD Biosciences, Cat#555485)<br>Viability dye (1:2000, eFluor 780, Thermo Fisher Scientific, Cat#65-0865-14)<br>TotalSeqTM-A cell hashing antibodies (1:500, Biolegend)
Validation	PE-Pan-CD11b: validated in splenocytes from BALB/c mice were stained with CD11b antibodies or with the corresponding REA Control antibodies.<br>APC-hCD45: validated on human peripheral blood lymphocytes, stained with either APC Mouse IgG1, or κ isotype control.<br>BV421-mCD45: validated on mouse splenic leucocytes preincubated with Purified Rat Anti-Mouse CD16/CD32 antibody and then stained with either BD Horizon™ BV421 Rat IgG2b or κ Isotype Control.

Eukaryotic cell lines

Policy information about cell lines and Sex and Gender in Research

Cell line source(s)	Name of line, Genotype, Source, Citation.<br>BIONi010-C-2, APOE e3/KO, Bioneer, EBiSC RRID:CVCL_II81.<br>BIONi010-C-3, APOE KO/KO, Bioneer, EBiSC RRID:CVCL_II82.<br>BIONi010-C-4, APOE e4/KO, Bioneer, EBiSC RRID:CVCL_II83.<br>BIONi010-C-6, APOE e2/KO, Bioneer, EBiSC RRID:CVCL_II85.
Authentication	The cell lines used here have been registered in the Human Pluripotent Stem Cell Registry, and has been fully characterized. BIONi010-C and subsequent genetic modifications - https://hpscreg.eu/cell-line/BIONi010-C. APOE genotypes were confirmed by Sanger sequencing and APOE knockout by Western blotting during the revision.
Mycoplasma contamination	All the lines used were regularly tested negative for mycoplams.
Commonly misidentified lines<br>(See ICLAC register)	None of the cell lines used in this study is known to be cross-contaminated or otherwise misidentified, and is not listed in the Register of Misidentified Cell Lines from ICLAC.

Animals and other research organisms

Policy information about studies involving animals; ARRIVE guidelines recommended for reporting animal research, and Sex and Gender in Research

Laboratory animals	AppNL-G-F Rag2-/- IL2rg-/- hCSF1-KI
Wild animals	No wild animals were used.
Reporting on sex	All animals were male.
Field-collected samples	No field-collected samples were used in this study.
Ethics oversight	Animal experiments were approved by the local Ethical Committee of Laboratory Animals of the KU Leuven (government licence LA1210579 ECD project number P177/2017) following local and EU guidelines.

Note that full information on the approval of the study protocol must also be provided in the manuscript.

Plants

Seed stocks	n/a
Novel plant genotypes	n/a
Authentication	n/a

Flow Cytometry

Plots

Confirm that:

- [x] The axis labels state the marker and fluorochrome used (e.g. CD4-FITC).
- [x] The axis scales are clearly visible. Include numbers along axes only for bottom left plot of group (a 'group' is an analysis of identical markers).
- [x] All plots are contour plots with outliers or pseudocolor plots.
- [x] A numerical value for number of cells or percentage (with statistics) is provided.

Methodology

Sample preparation	Mic were sacrificed with an overdose of sodium pentobarbital and immediately perfused with ice-cold 1x DPBS (Gibco, Cat#14190-144) supplemented with 5U of heparin (LEO). After perfusion, 1 hemisphere of each mouse brain without cerebellum and olfactory bulbs was placed in FACS buffer (1x DPBS, 2% FCS and 2 mM EDTA) + 5 µM Actinomycin D (ActD, Sigma, Cat#A1410-5MG) for transcriptomics. Brains were mechanically and enzymatically dissociated using Miltenyi neural tissue dissociation kit P (Miltenyi, Cat#130-092-628) supplemented with 5 µM ActD. Next, samples were passed through a 70 µm strainer (BD2 Falcon), washed in 10 ml of ice-cold FACS buffer + 5 µM ActD and spun at 300g for 15 minutes at 4°C. Note that 5 µM ActD was kept during collection and enzymatic dissociation of the tissue to prevent artificial activation of human microglia during the procedure as previously reported12. ActD was removed from the myelin removal step to prevent toxicity derived from long-term exposure. Following dissociation, myelin was removed by resuspending pelleted cells into 30% isotonic Percoll (GE Healthcare, Cat#17-5445-02) and centrifuging at 300g for 15 minutes at 4°C. Accumulating layers of myelin and cellular debris were discarded and Fc receptors were blocked in FcR blocking solution (1:10, Miltenyi, Cat#130-092-575) in cold FACS buffer for 10 minutes at 4°C. Next, cells were washed in 5 ml of FACS buffer and pelleted cells were incubated with the following antibodies: PE-Pan-CD11b (1:50, Miltenyi, Cat#130-113-806), BV421-mCD45 (1:500, BD Biosciences, Cat#563890), APC-hCD45 (1:50, BD Biosciences, Cat#555485), TotalSeqTM-A cell hashing antibodies (1:500, Biolegend) and viability dye (1:2000, eFluor 780, Thermo Fisher Scientific, Cat#65-0865-14) in cold FACS buffer during 30 minutes at 4°C. After incubation, cells were washed, and the pellet was resuspended in 500 µl of FACS buffer and passed through 35 µm strainer prior sorting.
Instrument	MACSQuant Tyto
Software	MACSQuantify™ Tyto® Software 1.0 and FCS express 7
Cell population abundance	Both mouse host (CD11b+ mCD45+) and human transplanted microglia (CD11b+ hCD45+) are two clear distinct populations. This study did not aim to quantify these populations by flow cytometry, but rather purify them for downstream trannscriptomic and epigenomic analyses.
Gating strategy	BSC and SSC were used to filter debri and doublet discrimination. e780 (Thermo Fisher) was used as a viability marker. All analyses were performed on viable singlets.

- [x] Tick this box to confirm that a figure exemplifying the gating strategy is provided in the Supplementary Information.
